# Physiological Notch Signaling Maintains Bone Homeostasis via RBPjk and Hey Upstream of NFATc1

**DOI:** 10.1371/journal.pgen.1002577

**Published:** 2012-03-22

**Authors:** Xiaolin Tu, Jianquan Chen, Joohyun Lim, Courtney M. Karner, Seung-Yon Lee, Julia Heisig, Cornelia Wiese, Kameswaran Surendran, Raphael Kopan, Manfred Gessler, Fanxin Long

**Affiliations:** 1 Department of Medicine, Washington University School of Medicine, St. Louis, Missouri, United States of America; 2 Developmental Biochemistry, Biocentre, University of Wuerzburg, Wuerzburg, Germany; 3 Department of Developmental Biology, Washington University School of Medicine, St. Louis, Missouri, United States of America; University of Illinois, United States of America

## Abstract

Notch signaling between neighboring cells controls many cell fate decisions in metazoans both during embryogenesis and in postnatal life. Previously, we uncovered a critical role for physiological Notch signaling in suppressing osteoblast differentiation in vivo. However, the contribution of individual Notch receptors and the downstream signaling mechanism have not been elucidated. Here we report that removal of Notch2, but not Notch1, from the embryonic limb mesenchyme markedly increased trabecular bone mass in adolescent mice. Deletion of the transcription factor RBPjk, a mediator of all canonical Notch signaling, in the mesenchymal progenitors but not the more mature osteoblast-lineage cells, caused a dramatic high-bone-mass phenotype characterized by increased osteoblast numbers, diminished bone marrow mesenchymal progenitor pool, and rapid age-dependent bone loss. Moreover, mice deficient in Hey1 and HeyL, two target genes of Notch-RBPjk signaling, exhibited high bone mass. Interestingly, Hey1 bound to and suppressed the NFATc1 promoter, and RBPjk deletion increased NFATc1 expression in bone. Finally, pharmacological inhibition of NFAT alleviated the high-bone-mass phenotype caused by RBPjk deletion. Thus, Notch-RBPjk signaling functions in part through Hey1-mediated inhibition of NFATc1 to suppress osteoblastogenesis, contributing to bone homeostasis in vivo.

## Author Summary

Osteoporosis is a disease caused by disruption of the balance between bone formation and resorption resulting in a net loss of bone mass. Although anti-resorptive agents are the current mainstay of osteoporosis therapy, novel strategies to promote bone formation are critically needed for more effective prevention and treatment of the disease. Notch signaling, an evolutionally conserved mechanism among multi-cellular organisms, was recently shown to control bone formation and therefore represents a potential target pathway for novel bone-promoting therapeutics. In this study we elucidate the intracellular signaling mechanism through which Notch controls bone formation, providing a molecular framework that may guide future drug development.

## Introduction

Notch signaling mediates communication between neighboring cells to control cell fate decisions in all metazoans [1], [2]. The mammalian genome encodes four Notch receptors (Notch1-4) and at least five ligands (Jagged1, 2 and Delta-like 1, 3, 4) [3]. In the canonical Notch pathway, binding of the ligands to the Notch receptors present on the neighboring cell surface triggers two successive intramembrane proteolytic cleavages of the receptors mediated by the γ-secretase complex and resulting in the release of the Notch intracellular domain (NICD) [4], [5], [6]. Upon its release from the plasma membrane, NICD translocates to the nucleus where it interacts with a transcription factor of the CSL family (RBPjk/CBF-1 in mammals) to activate transcription of target genes [7]. Among the best known targets of Notch/RBPjk signaling are the Hes/Hey family of basic helix-loop-helix (bHLH) transcription repressors [8]. However, the regulation of individual Hes/Hey proteins by Notch and their role in mediating Notch function are highly dependent on cell context. In addition to the canonical pathway, Notch has also been reported to signal through noncanonical, RBPjk-independent mechanisms, but the molecular nature of these mechanisms is not well understood [6], [9], [10], [11].

Notch signaling has emerged as a critical regulator of the mammalian skeleton. Initial mouse genetic studies identified a role for Notch in axial skeletal patterning, as mice lacking either Delta-like 3 (Dll3) [12], presenilin 1 (PS1) [13], [14], a catalytic subunit of the γ-secretase complex, or lunatic fringe, a glycosyltransferase that modifies Notch proteins [15], exhibited defects in the axial skeleton due to deficiency in somite segmentation and maintenance. In addition, mice lacking either Notch1 and 2 specifically in the limb bud ectoderm or Jagged2 globally displayed syndactyly [16], [17]. Consistent with the mouse studies, human mutations in Dll3 [18] were found to cause spondylocostal dysostosis, whereas those in Notch2 [10] and Jagged1 [19], [20] were responsible for Alagille syndrome.

More recent mouse genetic studies have expanded our view of Notch function in the osteoblast lineage. By genetically removing both catalytic subunits of the γ-secretase complex, PS1 and PS2, or both Notch1 and 2 in the embryonic limb mesenchyme, we have shown that Notch critically controls postnatal bone homeostasis: the Notch-deficient long bones exhibited excessive bone formation in adolescent mice with concomitant loss of bone marrow mesenchymal progenitors [21]. Consistent with the negative role of Notch in osteoblast differentiation, Zanotti et al reported that forced-expression of NICD in osteoblastic precursors reduced osteoblast numbers and caused osteopenia [22]. Conversely, forced-expression of NICD at a later stage of the osteoblast lineage led to sclerosis owing to excessive proliferation of the immature osteoblasts, highlighting stage-specific functions of constitutive Notch activation in the osteoblast lineage [23], [24]. The negative role of physiological Notch signaling in osteoblast differentiation uncovered in mice is congruent with the clinical findings that Notch1 haploinsufficiency causes ectopic osteoblast differentiation and calcification in the aortic valves [25], [26], whereas Notch2 stabilizing mutations are responsible for the Hadju-Cheney syndrome, a disorder of severe and progressive bone loss [27], [28]. However, the signaling cascade through which Notch inhibits osteoblastogenesis is not yet well understood.

Here we have genetically assessed the role of RBPjk and Hey proteins, known components of the Notch canonical pathway, in the regulation of osteoblastogenesis. Moreover, we have evaluated the role of NFAT in the high-bone-mass phenotype caused by RBPjk deficiency. The NFAT (nuclear factor of activated T cells) family of transcription factors (NFATc1-c4) [29] have been shown to play important roles in several skeletal cell types, including chondrocytes [30], osteoclasts [31] and osteoblasts [32], [33]. Our results support a model wherein canonical Notch signaling suppresses osteoblastogenesis in part through inhibition of NFATc1 transcription, therefore integrating extracellular signals with transcription factors that control osteoblast differentiation.

## Results

### Notch2 plays a dominant role in suppressing bone formation

**Figure 1:**
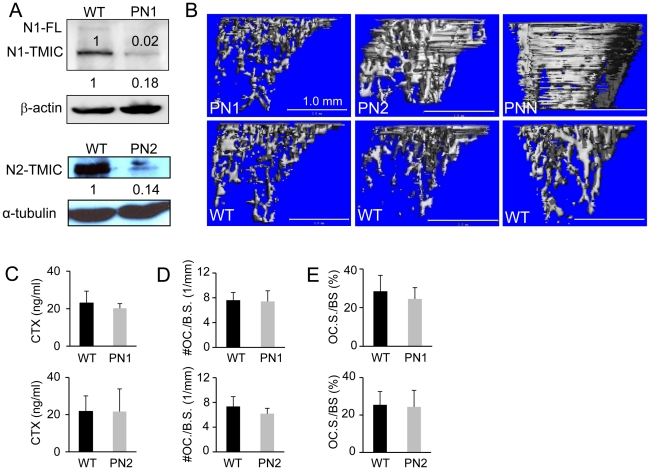
Bone phenotypes of PN1 and PN2 mice at 8 weeks of age. (A) Western blot analyses of Notch1 and 2 in PN1 and PN2 mice versus wild-type (WT) littermates. Protein extracts prepared from limb buds of E12.5 PN1 embryos or tibiae and femora of 8-week-old PN2 mice. Numbers indicate signal intensity relative to wild type (designated 1). N1-FL: full-length Notch 1; N1-TMIC, N2-TMIC: transmembrane-intracellular domain of Notch 1 and 2. Full-length N2 was not detected in either WT or PN2. (B) μCT three-dimensional reconstruction of metaphyseal trabecular bone of the tibia. (C) Serum CTX assays. (D) Number of osteoclasts normalized to trabecular bone perimeter (#OC./B.S. (1/mm)) on tibial sections. (E) Osteoclast surface normalized to bone surface (OC.S./B.S.) on tibial sections. Bar graphs show mean ± s.d., n = 3.

Previously, simultaneous removal of both Notch1 and 2 (PNN mice) from the embryonic limb mesenchyme with Prx1-Cre, which targets all of the early limb bud mesenchyme and a subset of the craniofacial mesenchyme [34], caused high bone mass due to increased osteoblast differentiation [21]. To discern the individual contributions of Notch1 versus 2 in the osteogenic progenitors, we employed the same Cre-loxP strategy to delete the two receptors separately. Western analyses confirmed that Notch1 or Notch2 was efficiently deleted in the limb mesenchyme of Prx1-Cre; Notch1^f/f^ (PN1) or Prx1-Cre; Notch2^f/f^ (PN2) mice, respectively (Figure 1A). As expected from our previous study of the PNN mice, PN1 and PN2 mice were viable without gross morphological anomalies. However, X-ray radiography of the limb bones at eight weeks of age revealed a marked increase in mineral content within the trabecular region of the PN2 but not the PN1 mice, when compared with their respective littermate controls (data not shown). Three-dimensional reconstruction using micro computed tomography (μCT) of the proximal tibial trabecular region confirmed this finding (Figure 1B). In particular, PN2 mice exhibited a 130% increase in trabecular bone volume owing to increased trabeculae numbers and decreased trabeculae spacing (Table 1). The PN2 phenotype was less dramatic than that of the PNN mice [21] (Figure 1B), indicating that Notch 1 performed a discernible role in the absence of Notch 2, even though deletion of Notch 1 alone did not cause an effect. Similar to the PNN mice, the high bone mass in PN2 mice was not due to decreased total osteoclast activity, as serum CTX levels, which reflect the amount of cleaved type I collagen by osteoclasts in the whole animal, did not differ significantly from the controls (Figure 1C). In addition, osteoclast number or osteoclast surface per bone perimeter did not change in either PN1 or PN2 mice (Figure 1D–1E). Therefore, physiological signaling from Notch 2, rather than Notch 1, plays a dominant role in suppressing bone formation.

**Table 1:** μCT analyses of PN1 and PN2 at 8 weeks of age.

	BV/TV			Tb.N*			Tb.Th*			Tb.Sp*		
Genotype	% (±s.d.)	Ratio over WT	p value	1/mm (±s.d.)	Ratio over WT	p value	mm (±s.d.)	Ratio over WT	p value	mm (±s.d.)	Ratio over WT	p value
PN1	13.087±6.413	1.3	0.498	2.827±0.295	1.2	0.137	0.068±0.016	1.0	0.910	0.368±0.043	0.8	0.168
WT	9.163±4.320			2.336±0.321			0.070±0.006			0.444±0.066		
PN2	21.343±2.788	2.3	0.004	3.471±0.262	1.6	0.015	0.072±0.017	1.2	0.568	0.298±0.032	0.6	0.023
WT	9.100±2.326			2.106±0.510			0.083±0.027			0.474±0.078		

BV: bone volume; TV; total volume; Tb.N*: trabeculae number; Tb.Th*: trabeculae thickness; Tb.Sp*: trabeculae spacing; data derived from 100 of 16-µm slices immediately below growth plate, n = 3 for each group. All analyses were performed with sex-matched littermates (all males for PN1, 1 male and 2 females for PN2).

### RBPjk mediates Notch function in suppressing osteoblast differentiation

**Figure 2:**
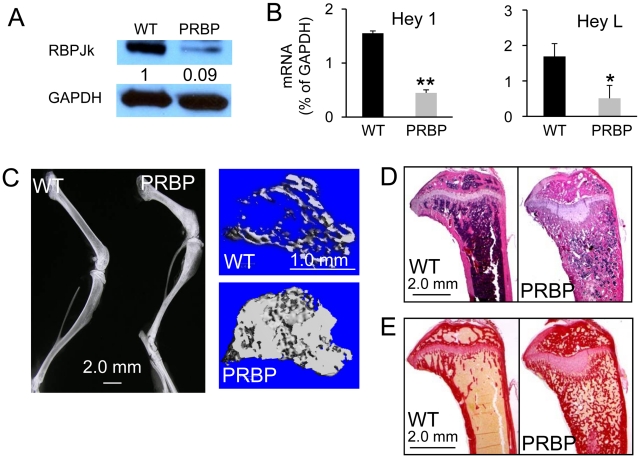
Bone mass of PRBP mice at 8 weeks of age. (A) Western analysis for RBPjk in protein extracts from tibiae and femora. (B) Real-time PCR with RNA from tibiae. (C) X-ray radiographs of hindlimbs (left), and μCT images of metaphyseal trabecular bone of the tibia (right). (D) H&E staining of medial longitudinal sections through the tibia. (E) Picrosirus red staining on medial longitudinal sections through the tibia. Collagen I stains red. Bar graphs show mean ± s.d., *p<0.05, **p<0.01, n = 3.

**Figure 3:**
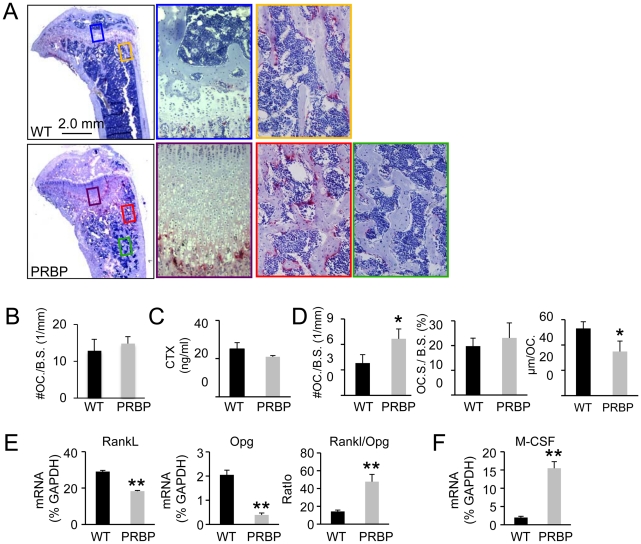
Osteoclasts in PRBP mice at 8 weeks of age. (A) TRAP staining on medial longitudinal sections through the tibia. Osteoclasts stain red. Color-coded boxed areas shown at higher magnification. (B) Osteoclast number normalized to cartilage perimeter at chondro-osseous junction (# OC. / B.S. (1/mm)). (C) Serum CTX assays. (D) Left to right: osteoclast number normalized to trabecular bone perimeter (# OC. /B.S. (1/mm)), osteoclast surface normalized to bone surface (OC. S. / B. S.), and average osteoclast surface (µm/OC.). (E–F) Real-time PCR with total RNA from tibiae. Bar graphs show mean ± s.d., *p<0.05, **p<0.01, n = 3.

To test the hypothesis that Notch suppresses bone formation through the canonical pathway, we deleted RBPjk with Prx1-Cre from the embryonic limb mesenchyme. Western analyses confirmed that RBPjk was efficiently deleted in the tibia of the Prx1-Cre; RBPjk^f/f^ (PRBP) mouse (Figure 2A). Moreover, Hey1 and HeyL, two Notch target genes previously identified in the PNN bones [21], were markedly reduced in the PRBP tibia (Figure 2B). The PRBP mice were born at mendelian ratio with no gross abnormalities. However, at eight weeks of age, X-ray radiography revealed that the PRBP mice contained much greater mineral content within the presumptive bone marrow cavity than the wild-type littermates (Figure 2C). μCT analysis of the proximal tibia confirmed a marked increase of bone mass in the PRBP mice (Figure 2C), as reflected in a 730%, 220% or 140% increase in BV/TV, trabeculae number or trabeculae thickness, respectively, coupled with a 70% decrease in trabeculae spacing (Table 2). Consistent with the μCT data, both H&E and picrosirius red staining of the tibia detected excessive trabecular bone occluding much of the marrow cavity of the PRBP bones (Figure 2D, 2E). These analyses also revealed an abnormal elongation of the growth plate hypertrophic cartilage in the PRBP bones (Figure 2D–2E); this phenotype was similar to that previously analyzed in the PNN mice and could not be contributed to changes in osteoclast numbers at the chondro-osseous junction (Figure 3A, 3B). The dramatic increase in bone mass in the PRBP mice was very similar to that seen in the presenilin 1- and 2-deficient (PPS) animals, but more severe than the PNN phenotype, likely due to contributions from Notch3 and 4 in the PNN mice [21]. Although the data do not exclude that RBPjk may control bone formation through a yet unknown mechanism independent of Notch, the striking similarity in the bone phenotype among the PPS, the PNN and the PRBP mice indicates that RBPjk is likely the principle mediator of physiological Notch signaling in bone.

**Table 2:** Age-dependent bone loss in PRBP mice.

		BV/TV			Tb.N*			Tb.Th*			Tb.Sp*		
Age	Genotype	% (±s.d.)	Ratio over WT	p value	1/mm (±s.d.)	Ratio over WT	p value	mm (±s.d.)	Ratio over WT	p value	mm (±s.d.)	Ratio over WT	p value
8 weeks	PRBP	47.217±11.535	8.3	0.003	5.683±0.980	3.2	0.002	0.1373±0.046	2.4	0.038	0.180±0.027	0.3	0.000
	WT	5.660±0.986			1.781±0.075			0.057±0.001			0.577±0.030		
26 weeks	PRBP	24.700±4.740	1.5	0.181	4.553±1.473	2.3	0.016	0.080±0.011	1.0	0.930	0.248±0.114	0.4	0.374
	WT	16.275±1.622			1.965±0.333			0.079±0.010			0.552±0.095		

BV: bone volume; TV; total volume; Tb.N*: trabeculae number; Tb.Th*: trabeculae thickness; Tb.Sp*: trabeculae spacing; data derived from 100 of 16-µm slices immediately below growth plate. All analyses were performed with sex-matched littermates (1 male and 2 females for 8 weeks, 2 males and 2 females for 26 weeks).

We then analyzed the cellular basis for the high bone mass in the eight-week-old PRBP mice. Tartrate-resistant acid phosphatase (TRAP) staining on tibial sections revealed a strikingly uneven distribution of osteoclasts within the trabecular bone region of the PRBP mice: whereas TRAP-positive cells were more abundant than normal within the metaphyseal region, few were detected towards the diaphysis (Figure 3A). The reason for this regional disparity is not certain at present but may be due to uneven compartmentalization of osteoclast precursors within the occluded marrow cavity. Serum CTX assay did not detect any significant difference between the PRBP and the WT littermates (Figure 3C). Further investigation of the metaphyseal region revealed that although osteoclast number per bone perimeter (No. OC./mm) was higher in the PRBP mice, the spreading of individual osteoclasts (µm/OC.) was decreased, resulting in no change in the percentage of bone surface covered by osteoclasts (OC. S./B. S.) (Figure 3D). Thus, the PRBP mice possessed abundant, but apparently less functional osteoclasts within the metaphyseal trabecular bone. Real-time PCR experiments showed that the mRNA levels for both the osteoclastogenic signal Rankl and the anti-osteoclastogenic factor Opg were reduced in the PRBP bone, but the ratio of Rankl over Opg (Rankl/Opg) was 230% higher in the PRBP bone than the control (Figure 3E). Moreover, the mRNA level for M-CSF, a potent mitogen of osteoclast precursors, was 690% higher in the PRBP mice (Figure 3F). The higher level of M-CSF coupled with an increased ratio of Rankl/Opg could explain the supernumerary but dysfunctional osteoclasts populating the metaphyseal trabecular bone in the PRBP mice. Overall, the high bone mass in the PRBP mice was not caused by an overall decrease in bone resorption.

**Figure 4:**
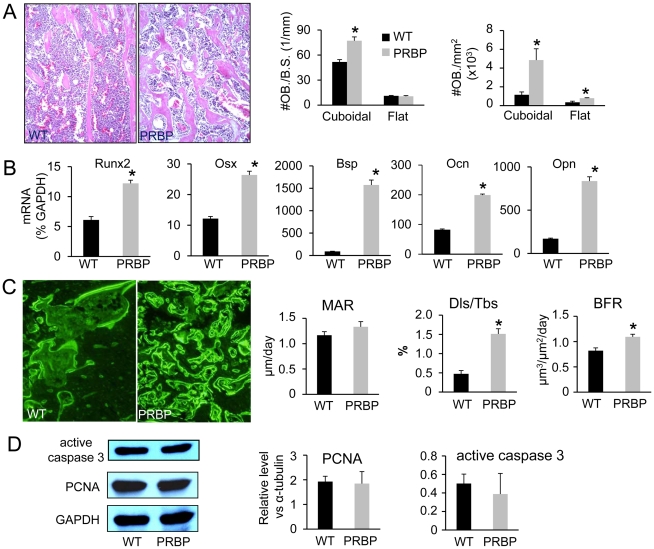
Osteoblasts in PRBP mice at 8 weeks of age. (A) Left to right: images of the trabecular bone region, osteoblast numbers normalized to trabecular bone perimeter (#OB./mm) and area (#OB/mm^2^) on sections. (B) Real-time PCR of bone total RNA. Osx: osterix; Bsp: bone sialoprotein; Ocn: osteocalcin; Opn: osteopontin. (C) Left to right: images of calcein-double-labeled trabecular bone region; mineal apposite rate (MAR), double-labeled surface over total bone surface (Dls/Tbs) and bone formation rate (BFR) in trabecular bone region. (D) Left to right: images of representative Western blots with protein extracts from tibiae and femora; quantifications of Western analyses for PCNA and active caspase 3. Bar graphs show mean ± s. d., *p<0.05, n = 3 (A–C) or 5 (D).

Having ruled out resorption deficiency as the main cause for the high bone mass in PRBP mice, we next focused on bone formation parameters. Static histomorphometry of tibial sections from the eight-week-old PRBP mice revealed a marked increase in the number of cuboidal (active) osteoblasts, when normalized to either bone perimeter (60% increase) or trabecular bone area (400% increase) (Figure 4A). The number of flat (inactive) osteoblasts, when normalized to trabecular bone area, was also increased by 100% in the PRBP mice. Consistent with the increase in osteoblast number, real-time PCR experiments showed that a number of common osteoblast markers were upregulated in bone total RNA (Figure 4B). Dynamic histomorphometry showed that the mineral apposition rate (MAR), which measured osteoblast activity, did not differ significantly between PRBP and the control littermates (Figure 4C). However, the percentage of double-labeled trabcular bone surface was increased by 230% in the PRBP mice, resulting in a significant increase in the bone formation rate (BFR) within the trabecular region (Figure 4C). Thus, the increase in bone mass in the PRBP mice was primarily due to a marked increase in osteoblast numbers.

To explore the mechanism responsible for the increase in osteoblast numbers, we assessed the status of apoptosis and proliferation of osteoblasts in PRBP versus wild-type bones. To this end, osteoblast protein extracts were prepared from the bone surface of the long bones, and subjected to Western analyses for activated caspase 3 and PCNA, markers for apoptosis and cell proliferation, respectively. These assays did not detect a significant difference in either protein between the genotypes (Figure 4D). Therefore, the increase in osteoblast numbers is unlikely to be caused by changes in apoptosis or proliferation, but rather due to enhanced differentiation from the progenitors.

### RBPjk deletion causes diminution of bone marrow mesenchymal progenitor pool and rapid age-dependent bone loss

**Figure 5:**
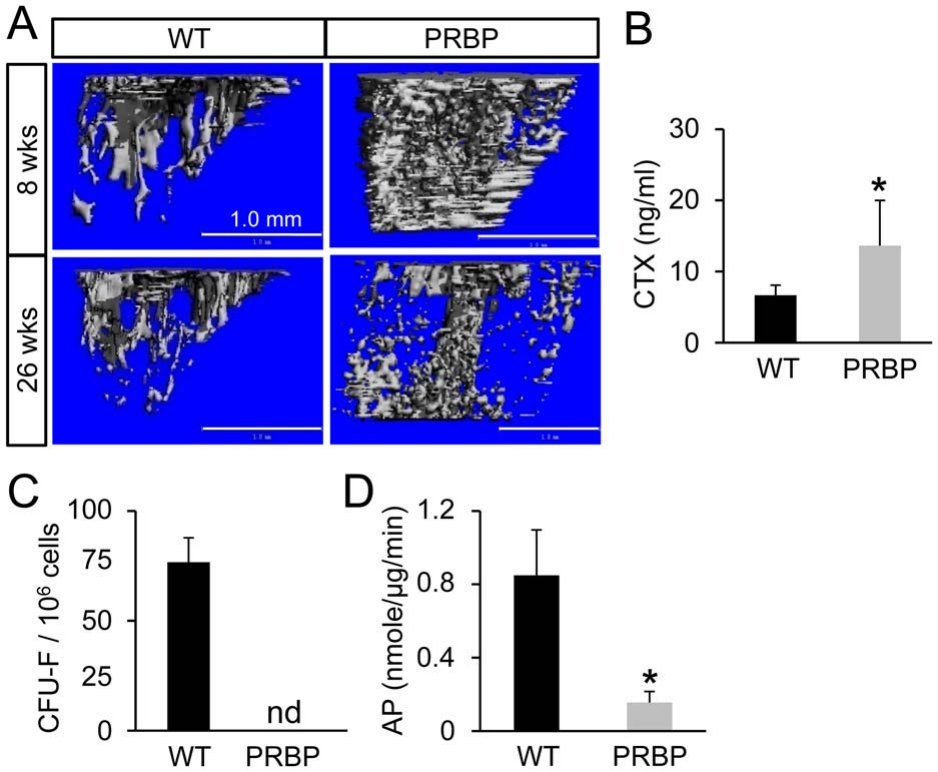
Bone loss in PRBP mice at 26 weeks of age. (A) μCT reconstruction of metaphyseal trabecular bone of tibia in wild type (WT) and PRBP mice. (B) Serum CTX assays. (C) Bone marrow CFU-F assays. (D) Alkaline phosphatase (AP) assays of BMSC in osteogenic medium. Bar graphs show mean ± s. d., *p<0.05, n = 3.

Uncontrolled osteoblast differentiation may lead to loss of bone marrow mesenchymal progenitors and rapid age-dependent boss loss [21]. To test whether this is the case in the PRBP mice, we analyzed bone mass by X-ray (data not shown) and μCT at 26 weeks of age. Indeed, bone mass was drastically reduced in the PRBP mice at 26 weeks when compared with 8 weeks (Figure 5A). When quantified, the trabecular bone mass of the PRBP tibia was no longer significantly different from the wild type at 26 weeks, representing a drastic decline from a level 730% above normal at 8 weeks (Table 2). Similarly, both trabeculae thickness and number were reduced to levels either equivalent or close to the wild type. Interestingly, bone resorption, as measured by serum CTX assays, was significantly higher in the PRBP mice over the control at 26 weeks, likely contributing to the rapid bone loss (Figure 5B). Thus,similar to the PNN mice [21], the PRBP mice, despite their high bone mass when young, rapidly lost bone with age.

We next assessed the status of the mesenchymal progenitors in the bone marrow. To this end, bone marrow stromal cells (BMSC) isolated from PRBP versus wild type littermates were subjected to CFU-F (colony forming unit-fibroblast) assays. These assays were not feasible with adolescent PRBP mice due to the occlusion of the marrow cavity, and therefore performed only after six months of age. Remarkably, no type I CFU-Fs could be detected from the PRBP bone marrow at either 26 weeks (data not shown) or one year (Figure 5C), indicating a severe diminution of the mesenchymal progenitor pool. Moreover, BMSC isolated from the PRBP bone were severely deficient in undergoing osteoblast differentiation when cultured in osteogenic media and monitored by the expression of alkaline phosphatase (AP) (Figure 5D). Therefore, the PRBP animals exhibited a marked deficiency in the bone marrow mesenchymal progenitor pool.

### RBPjk is not critical for later stages of osteoblast lineage

**Figure 6:**
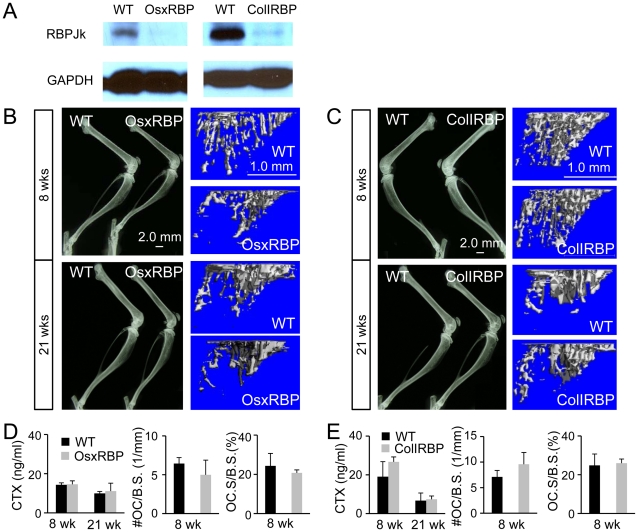
Deletion of RBPjk at later stages of osteoblast lineage. (A) Western analyses of RBPjk in protein extracts from tibiae and femora from OsxRBP and ColIRBP versus wild type (WT) littermates at 8 weeks of age. (B–C) X-ray radiographs of hindlimbs, and μCT reconstruction of tibia metaphyseal trabecular region at 8 and 21 weeks of age. (D–E) Osteoclast parameters of OsxRBP and ColIRBP at 8 and 21 weeks of age. Bar graphs show mean ± s. d., n = 3.

To delineate potential stage-specific requirement of Notch-RBPjk signaling during osteoblast differentiation, we deleted RBPjk with either Osx-GFP::Cre [35] or 2.3ColI-Cre [36], which are believed to target progressively more mature osteoblastic cells. Western analyses confirmed that both Cre lines efficiently deleted RBPjk in the long bones (Figure 6A). However, when assessed by either X-ray radiography or μCT, neither deletion caused any significant changes in bone mass at either 8 or 21 weeks of age (Figure 6B, 6C), a finding confirmed by quantitative analyses (Table 3). Because previous studies have suggested that Notch signaling in the more mature osteoblastic cells regulated osteoclastogenesis through modulation of Rankl and Opg [23], [37], we examined osteoclasts in both the Osx-GFP::Cre; RBPjk^f/f^ (OsxRBP) and the 2.3ColI-Cre; RBPjk^f/f^ (ColIRBP) mice. However, serum CTX assays detected no significant changes in either OsxRBP or ColIRBP mice over controls at either 8 or 21 weeks of age (Figure 6D, 6E), indicating largely normal bone resorption in these animals. Similarly, osteoclast number and osteoclast surface per bone surface were comparable between the mutant strains and their wild-type littermates (Figure 6D, 6E). Thus, RBPjk does not appear to play a major role in the more committed osteoblast-lineage cells.

**Table 3:** μCT analyses of OsxRBP and ColIRBP at 8 and 21 weeks of age.

		BV/TV			Tb.N*			Tb.Th*			Tb.Sp*		
Age	Genotype	% (±s.d.)	Ratio over WT	p value	1/mm (±s.d.)	Ratio over WT	p value	mm (±s.d.)	Ratio over WT	p value	mm (±s.d.)	Ratio over WT	p value
8 weeks	OsxRBP	5.150±2.401	0.7	0.492	2.710±0.492	1.4	0.083	0.052±0.004	1.0	0.936	0.374±0.077	0.7	0.082
	WT	7.045±4.587			1.887±0.377			0.051±0.002			0.561±0.116		
21 weeks	OsxRBP	7.513±0.792	0.8	0.400	1.904±0.804	1.2	0.464	0.060±0.005	0.8	0.069	0.582±0.211	0.8	0.262
	WT	8.923±2.480			1.530±0.167			0.073±0.007			0.701±0.086		
8 weeks	ColIRBP	6.003±1.908	1.2	0.724	2.230±0.556	1.1	0.771	0.056±0.008	1.1	0.406	0.451±0.096	0.8	0.550
	WT	5.187±3.216			2.135±0.865			0.051±0.003			0.532±0.194		
21 weeks	ColIRBP	8.025±1.024	0.8	0.394	1.700±0.063	1.0	0.693	0.069±0.002	0.9	0.172	0.609±0.024	1.0	0.706
	WT	9.533±0.794			1.635±0.273			0.076±0.006			0.630±0.198		

BV: bone volume; TV; total volume; Tb.N*: trabeculae number; Tb.Th*: trabeculae thickness; Tb.Sp*: trabeculae spacing; data derived from 100 of 16-µm slices immediately below growth plate. All analyses performed with sex-matched littermates (3 females for ColIRBP at either 8 or 21 weeks, 2 males and 2 females for OsxRBP at 8 weeks, 1 male and 2 females for OsxRBP at 21 weeks).

### Hey1 and HeyL mediate Notch-RBPjk signaling in osteoblast lineage

**Figure 7:**
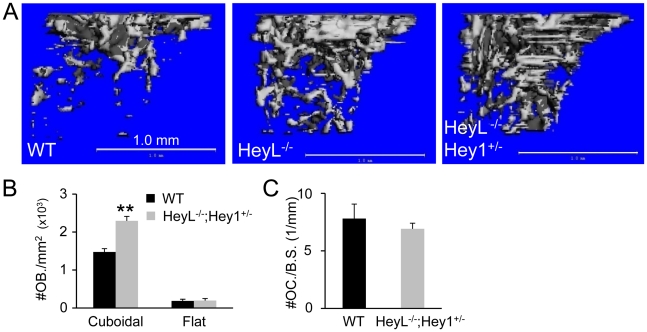
Bone phenotypes of Hey1 and HeyL mutant mice at 8 weeks of age. (A) μCT 3-D reconstruction of metaphyseal trabecular bone of the femur from WT, HeyL^−/−^, and HeyL^−/−^; Hey1^+/−^. (B) Number of osteoblasts per trabecular bone area on sections (No. OB/mm^2^). (C) Number of osteoclasts normalized to trabecular bone perimeter (No. OC/mm). Bar graphs show mean ± s. d., ** p<0.01, n = 4.

To assess the role of Hey1 and HeyL in bone formation, we analyzed the bones of mice wherein the two genes have been deleted. Because previous work by others revealed no major bone phenotype in the Hey1^−/−^ mice [38], we focused on the HeyL^−/−^ and the Hey1/HeyL double mutant animals, all in the C57BL6 background. As Hey1^−/−^; HeyL^−/−^ mice died prematurely due to heart defects [39], we analyzed the bones of the viable HeyL^−/−^; Hey1^+/−^ animals. μCT analyses showed that the HeyL^−/−^ and the HeyL^−/−^; Hey1^+/−^ mice possessed progressively more trabecular bone than their wild-type littermates at 8 weeks of age (Figure 7A). In particular, the femoral trabecular bone mass was increased by 80% and 150% over the control in the HeyL^−/−^ and the HeyL^−/−^; Hey1^+/−^ animals, respectively (Table 4). Moreover, like the PRBP bones, the HeyL^−/−^; Hey1^+/−^ samples exhibited a significant increase in trabeculae number and thickness with a corresponding decrease in trabeculae spacing. At the cellular level, the HeyL^−/−^; Hey1^+/−^ bones exhibited more cuboidal osteoblasts than the wild type whereas their number of osteoclasts appeared to be normal (Figure 7B, 7C). Thus, Hey1 and HeyL, like Notch and RBPjk, negatively regulate osteoblast numbers.

**Table 4:** μCT analyses of HeyL^−/−^; Hey1^+/−^ and HeyL^−/−^ at 8 weeks of age.

	BV/TV			Tb. N*			Tb.Th*			Tb.Sp*		
Genotype	% (±s.d.)	Ratio over WT	p value	1/mm (±s.d.)	Ratio over WT	p value	mm (±s.d.)	Ratio over WT	p value	mm (±s.d.)	Ratio over WT	p value
HeyL−/−	17.027±2.818	1.8	0.006	2.410±0.321	1.1	0.653	0.101±0.003	1.2	0.129	0.456±0.074	0.9	0.728
HeyL−/−; Hey1+/−	23.254±4.123	2.5	0.000	3.295±0.480	1.5	0.014	0.107±0.009	1.3	0.027	0.344±0.048	0.7	0.036
WT	9.320±1.675			2.247±0.477			0.081±0.018			0.483±0.109		

BV: bone volume; TV; total volume; Tb.N*: trabeculae number; Tb.Th*: trabeculae thickness; Tb.Sp*: trabeculae spacing. Data derived from 100 of 16-mm slices immediately below growth plate, n = 4 for each group (all females in C57BL6 background).

### NFATc1 functions downstream of Notch-RBPjk-Hey signaling

**Figure 8:**
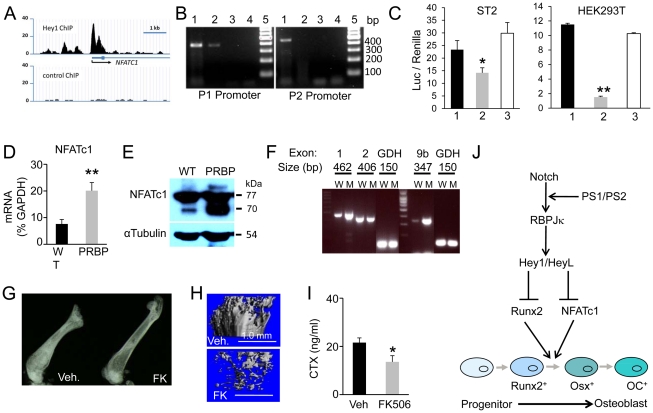
Relationship between Notch-RBPjk and NFAT in bone. (A) ChIP-seq data showing Flag-Hey1 binding at NFATc1 P1 promoter region in HEK293T cells. (B) ChIP showing Flag-Hey1 bound to P1 but not P2 promoter of NFATc1 in ST2 cells. 1: input; 2: Flag antibody; 3: IgG; 4: water control; 5: molecular weight ladder. (C) NFATc1 P1 promoter luciferase reporter assays in ST2 and HEK293T cells. 1: co-transfection with pCS2; 2: co-transfection with pCS2-Hey1; 3: co-transfection with pCS2-Hey1-ΔHLH. (D) NFATc1 real-time PCR with total RNA from tibiae of 8-week-old mice. (E) Western blot analysis of NFATc1 in protein extracts from tibiae and femora at 8 weeks. (F) Exon-specific RT-PCR of NFATc1 mRNA variants in bone RNA from 8-week-old mice. W: wild type mice; M: PRBP mice; GDH: glyceraldehyde 3-phosphate dehydrogenase. (G) X-ray radiographs of femora from PRBP mice treated with vehicle (Veh.) or FK506 (FK). (H) μCT 3-D reconstruction of the femur metaphyseal trabecular region. (I) Serum CTX assays. (J) A model for Notch signaling in regulating osteoblast differentiation. ↓: stimulation; ⊥: negative regulation. Bar graphs show mean ± s. d., * p<0.05, ** p<0.01, n = 3.

We next investigated the mechanism through which Notch-RBPjk-Hey signaling regulates osteoblast differentiation. In a separate effort to identify Hey1 and HeyL target genes, we performed genome-wide ChIP-seq (Chromatin immunoprecipitation followed by high-throughput sequencing) experiments by expressing Flag-tagged Hey1 or HeyL in HEK293 cells. We identified strong binding for both proteins around the P1 promoter of NFATc1 (Figure 8A and data not shown). Importantly, Hey1 was also found to bind to the NFATc1 P1 promoter region in ST2 cells, a mouse bone marrow stromal cell line that can be induced to differentiate into osteoblasts (Figure 8B); the binding is consistent with the presence of a predicted Hey1 binding site “CGCGCG” within the region. In contrast, no binding was detected for the alternative P2 promoter (Figure 8B). We next focused on the functional relevance of Hey1 binding. Full-length Hey1, but not a form missing the HLH domain, suppressed the activity of the NFATc1 P1 promoter in transient transfection assays in both HEK293T and ST2 cells (Figure 8C). Because NFATc1 was previously shown to increase osteoblast numbers [33], we explored the potential involvement of NFATc1 in Notch-RBPjk signaling in bone. Real-time PCR revealed that NFATc1 mRNA was increased by 200% in the PRBP tibia over the control (Figure 8D). Western analyses identified a 70 kD isoform of NFATc1 greatly induced in the PRBP bones, whereas a 77 kD form was less affected (Figure 8E). To gain insight on the induced isoform, we employed semi-quantitative RT-PCR to identify the specific NFATc1 mRNA variant(s) increased in the PRBP bones. The NFATc1 mRNA variants are known to differ both at the 5′ end containing either exon1 or 2, and at the 3′ end that either terminates with exon 9b, or contains exon 9a through exon 11. By using primer pairs spanning exons 1 and 3, 2 and 3, 8 and 9b or 8 and 11, we observed a marked increase of exon 1 in the PRBP samples, whereas exon 2 was unchanged (Figure 8F). Moreover, exon 9b was enriched in PRBP over wild type, whereas exon 11 was not detectable in either genotype (Figure 8F, and data not shown). Thus, an NFATc1 mRNA variant transcribed from the P1 promoter and containing exons 1 and 9b was specifically induced in the PRBP bones.

The finding above raises the possibility that the increase in NFATc1 might contribute to the high bone mass in the PRBP mice, and further that inhibition of NFATc1 activity may be able to alleviate the phenotype. To test this hypothesis, we injected littermate PRBP animals daily with either FK506, a potent inhibitor of NFAT signaling, or vehicle, for one month starting at one month of age. As expected, the vehicle treatment did not alter the high bone mass phenotype of the PRBP animals. However, FK506 markedly reduced bone mass in the PRBP mice, especially in the femur (Figure 8G and 8H, Table 5). This reversal of the high bone mass occurred in the face of decreased bone resorption in the FK506-treated animals, as indicated by a significantly lower serum CTX level (Figure 8I), presumably due to the known role of NFAT in osteoclastogenesis [31]. The suppression of bone resorption may explain the observation that FK506 did not consistently correct the high bone mass in the tibia (data not shown). However, in one case where the serum CTX level was less affected by FK506, both the tibia and the femur were corrected (Figure S1). As controls, the wild-type littermates were subjected to the same inhibitor or vehicle treatment. Similar to a previous report [32], the trabecular bone mass was reduced in the wild-type animals by FK506, but the extent of reduction was modest compared to that seen in the PRBP mice (Figure S2). Overall, the results showed that NFAT inhibition could override the effect of RBPjk deletion on bone mass. Furthermore, because Hey1 directly inhibits NFATc1 expression, Notch-RBPjk-Hey signaling appears to inhibit bone formation in part by down-regulating NFATc1.

**Table 5:** μCT analyses of FK-506-injected PRBP mice.

	BV/TV			Tb.N*			Tb.Th*			Tb.Sp*		
	% (±s.d.)	Ratio over vehicle	p value	1/mm (±s.d.)	Ratio over vehicle	p value	mm (±s.d.)	Ratio over vehicle	p value	mm (±s.d.)	Ratio over vehicle	p value
FK506	4.078±3.585	0.14	0.001	2.265±1.073	0.63	0.207	0.051±0.003	0.42	0.003	0.503±0.167	1.45	0.182
Vehicle	30.138±7.444			3.594±1.540			0.121±0.029			0.346±0.124		

BV: bone volume; TV; total volume; Tb. N*: trabeculae number; Tb. Th*: trabeculae thickness; Tb. Sp*: trabeculae spacing; data derived from 100 of 16-µm slices immediately below growth plate, n = 4 for each group, all analyses done with sex-matched littermates (3 females and 1 male).

## Discussion

The present study establishes canonical Notch signaling as a critical mechanism for maintaining bone homeostasis under normal physiological conditions. In this capacity, Notch appears to function as a gatekeeper to ensure that a proper number of osteoblasts are produced during differentiation. Because removal of Notch signaling from the Osx-positive stage onward did not have an obvious effect, we propose that Notch mainly controls the transition from Runx2- to Osx-positive cells. Mechanistically, Notch signals through RBPjk to induce transcription of Hey1 and HeyL, which in turn inhibit osteoblast differentiation by suppressing both Runx2 activity [21] and NFATc1 expression (this study) (Figure 8J).

The current study not only demonstrates the stage- and receptor-specificity of Notch signaling during osteoblast differentiation, but also sheds light on the intracellular mechanism mediating Notch function. Although RBPjk was previously shown to mediate the effect of NICD overexpression on both chondrogenesis and preosteoblast proliferation [24], [40], this study establishes for the first time the importance of RBPjk in physiological Notch signaling within the osteoblast lineage. Moreover, the present study uncovers a direct regulation of NFATc1 expression by Notch signaling.

The relationship between Notch and NFAT signaling warrants further investigation. The direct suppression of NFATc1 promoter by Hey1, and the dominant effect of FK506 over RBPjk removal support the model wherein NFAT functions downstream of and opposite to Notch signaling in regulating bone formation. However, we cannot rule out that FK506 may have NFAT-independent functions, or that the systemically-delivered FK506 acted on other cell types to affect bone mass indirectly. Our finding that Hey1 binds to and suppresses the NFATc1 promoter is consistent with a recent report that Notch inhibited NFATc1 transcription in ST2 and primary osteoblasts [41]. However, NFATc1 is unlikely to be the sole effector, as simultaneous removal of NFATc1 and RBPjk with Prx1-Cre did not rescue the high bone mass phenotype caused by RBPjk deletion (data not shown). NFATc2 may play a redundant role as it was previously shown to stimulate osteoblast differentiation [32]. Indeed, Western blotting showed that several isoforms of NFATc2 were markedly increased in the PRBP bones over the control (Figure S3). The mechanism for this upregulation however, is currently unknown. Future experiments with simultaneous deletion of NFATc1 and NFATc2 will test the hypothesis that the two proteins redundantly mediate Notch function in bone.

Notch-RBPjk removal from the early limb mesenchyme led to a severe deficit in type I CFU-F in the postnatal bone marrow. This phenotype could reflect either a direct requirement of Notch signaling in the CFU-F cells, or a secondary effect due to changes in osteoblast differentiation. To distinguish these possibilities, we performed lineage-tracing experiments with the Rosa26 reporter mouse, and observed that the type I CFU-F cells were not targeted by Prx1-Cre although some stromal cells in the same culture were (data not shown). In addition, experiments with a transgenic mouse (TNR) that reports Notch-RBPjk signaling [42] revealed that some stromal cells but not the type I CFU-F cells exhibited canonical Notch signaling *in vitro* (data not shown). Thus, diminution of bone marrow mesenchymal progenitors, as reflected by CFU-F assays *in vitro*, was likely to be secondary to changes in osteoblast differentiation. In this scenario, assuming mesenchymal progenitors normally exist in equilibrium with Runx2-positive osteogenic precursors, we envision that unchecked differentiation of the latter due to Notch deficiency may lead to exodus of cells from the mesenchymal progenitor pool. Alternatively, the altered bone marrow environment due to the excessive bone mass in Notch-deficient mice may be unfavorable for either establishing or maintaining a normal mesenchymal progenitor pool.

A mechanistic understanding of the dominance of Notch2 over Notch1 awaits further studies. A similar dominance of Notch2 over 1 was observed during nephron formation in the mouse embryo [43], [44], whereas a dominance of Notch1 over 2 was reported in the skin [45], [46] as well as in osteoclasts [37]. The mechanism underlying the differential roles among Notch paralogs is currently unclear, but it could reflect differences in either expression levels or ligand-binding affinities among the different receptors within a given cell type. This model predicts that Notch2 is normally preferably activated in the osteogenic progenitors. Alternatively, Notch1 and2 may be similarly activated but Notch2-NICD is more potent than Notch1-NICD in suppressing osteoblast differentiation. However, Notch 1 deficiency was sufficient to cause ectopic ossification in human aortic valves [25]. Thus, the relative contribution of Notch1 versus 2 in suppressing the osteogenic program appears to be context dependent.

The effect of Notch signaling in osteoblast-lineage cells on osteoclast differentiation is likely to be complex. Although TRAP-positive osteoclasts were more abundant than normal within the metaphyseal trabecular region of the PRBP bones, the total bone resorption activity was relatively normal at 8 weeks of age. However, by 26 weeks bone resorption was more robust in the PRBP mice than the littermate control, and likely contributed to the rapid bone loss seen at this age. The mechanism for the age-dependent bone resorption phenotype is not understood at present, but likely involves additional factors beyond Notch deficiency in the osteoblast lineage. In addition, unlike the PRBP mice, the PN1, PN2, OsxRBP and ColIRBP mice did not display an obvious bone resorption phenotype at either 8 or 21 weeks, even though certain changes in M-CSF, Rankl and Opg were observed in these animals (Figure S4). The lack of bone resorption phenotype in the ColIRBP mice appears to be at odds with the previous report that deletion of presenilin 1 and 2 by 2.3Col1-Cre caused an increase in bone resorption at six months, although not at three months of age [23]. Besides trivial explanations such as slight age differences or genetic background variations between the two studies, the discrepancy could indicate that the previously observed effect was independent of RBPjk. In summary, the predominant function of physiologic canonical Notch signaling in the osteoblast lineage is suppression of osteoblastogenesis from the precursors. Thus, potential pharmaceutical inhibition of this pathway in osteogenic progenitors may be beneficial for bone formation.

## Materials and Methods

### Mouse strains

The *N1^f/f^* [47], *N2^f/f^* [48], *RBPjk^f/f^* [49], *2.3ColI-Cre* [36], *Osx-GFP::Cre* [35], *Prx1-Cre* [34], *Hey1^+/−^* [50], *HeyL^−/−^* [39] and *NFATc1^f/f^* [51] mouse strains are as previously described. The Animal Studies Committee at Washington University approved all mouse procedures.

### Analyses of mice

Radiographs of mouse skeleton were generated using a Faxitron X-ray system (Faxitron X-ray Corp) with 20-second exposure under 25 kV. Micro computed tomography (μCT 40, Scanco Medical AG) was used for three-dimensional reconstruction, and quantification of bone parameters (threshold set at 200). Serum CTX assays were conducted with mice without feeding for 6 hours with the RatLaps ELISA kit (Immunodiagnostic Systems Ltd.). H&E, TRAP and picro-sirius red staining were performed on paraffin sections, following decalcification for postnatal samples. For dynamic histomorphometry of postnatal mice, calcein (Sigma) was injected intraparitoneally at 7.5 mg/kg on days 7 and 2 prior to sacrifice, and bones were sectioned in methyl-methacrylate. Bioquant II was used for quantification in both static and dynamic bone histomophometry. FK506 (Sigma) was dissolved in DMSO and was injected subcutaneously into one-month-old mice at 0.30 mg/kg/day for one month before harvest.

### Cell cultures, transfections, and analyses of protein and RNA

The CFU-F and osteoblast differentiation assays were preformed as previously described [21]. Only type I CFU-Fs were scored in the present study.

Transient transfections were performed as follows. ST2 were plated at 3×10^4^/well in a 24-well plate overnight, and transfected with pCS2-Hey1, pCS2-Hey1-ΔHLH or empty pCS2 vector (0.2 µg) [21], pNFATc1-0.8P1 (0.1 µg) [52] and pRL-Renilla (0.01 µg, Promega) for 8 h using Lipofectamine (1 µl/well). HEK293T cells were plated at 4×10^5^/well in a 12-well plate overnight, transfected with pCS2-Hey1, pCS2-Hey1-ΔHLH or empty pCS2 vector (0.4 µg), pNFATc1-0.8P1 (0.2 µg) and pRL-Renilla (0.02 µg) using Fugene (1.8 µl/well). The transfected ST2 or HEK293T cells were harvested at 48 hours after the beginning of transfection and subjected to dual luciferase activity assays (Promega).

Western analyses were performed with bone proteins extracted with RIPA buffer from tibiae and femora that were cut into small pieces after bone marrow cells were flushed out. The Notch 1 monoclonal antibody mN1A was as previously described [53], and the Notch 2 antibody (C651.6DbHN) was from Developmental Studies Hybridoma Bank at the University of Iowa. The antibody against RBPjk was from Cosmobio (Japan).

Real time PCR was performed with SYBR-Green (Roche) in ABI-7500 (Applied Biosystems) using cDNA reverse-transcribed from bone total RNA, extracted with Trizol (Invitrogen) from pulverized tibia and femur after removal of the bone marrow. Sequence information for the real-time PCR primers is listed in Table S1. The exon-specific primers for NFATc1 (Table S2) were as previously described [54], but the exons were renumbered according to the current NCBI nucleotide database. Semi-quantitative RT-PCR for NFATc1 was performed at an annealing temperature of 57°C for 40 cycles. GAPDH used as loading control was amplified for 30 cycles.

### ChIP experiment

ST2 cells were infected with lentivirus to express a doxycycline-inducible Flag-Hey1 transgene. Flag-Hey1 expression was induced with100 ng/mL Doxycycline (Sigma D9891) for 12 hours. Chromatin and protein complexes were crosslinked for 10 minutes in 1% formaldehyde and flash frozen. The chromatin was sonicated to an average size of 200–450 bp using a Sonics Vibracell sonicator (model Vcx 500). Chromatin complexes were immunoprecipitated using an anti-Flag antibody (Sigma F1804). Immunoprecipitated DNA fragments were amplified by PCR using primers adjacent to a predicted Hey1-binding site within the P1 promoter of the mouse NFATc1 gene (5′ - TCTCGGTCTCACTCTGACGCA - 3′ and 5′ - TTCCCTCTTGTACACCTTTGCCCA - 3′), or primers near the P2 promoter approximately 4 Kb downstream (5′ - TCCGGGTTTACATAAACAAGCGGC - 3′ and 5′ - ACTGCACACCACGCTGAACAGGAA - 3′).

### ChIP–seq experiment

HEK293 cells that express a doxycycline-regulated Flag-Hey1 or -HeyL transgene were used for ChIP-seq analysis. Cells were induced with 50 ng/ml doxycycline for 48 hours to ensure a low-level expression and Hey1- or HeyL-containing chromatin was immunoprecipitated with a Flag antibody. Cells carrying the same transgenes but grown without doxycycline were used as control. Preparation of ChIP libraries, Illumina sequencing and data analysis were performed as previously described [55].

## Supporting Information

Figure S1 Correction of the high-bone-mass phenotype in the tibia by FK506. (A) X-ray radiographs of the tibia. Note a notable correction of the shape of the tibia. (B) Data from μCT analyses and serum CTX assays of animals in (A).
(TIF)

Figure S2 Effect of FK506 on trabecular bone mass of wild type mice. Shown are μCT 3-D reconstruction images of the metaphyseal trabecular region of the tibia.
(TIF)

Figure S3 Western analyses of NFATc2 in protein extracts from tibiae and femora of 8-week-old PRBP versus wild-type littermates.
(TIF)

Figure S4 Real-time PCR of osteoclastogenic factors in bone RNA from indicated mouse strains. Values are normalized to wild type levels (designated 1). Bar graphs show mean ± s. d., *p<0.05, **p<0.01, n = 3.
(TIF)

Table S1 Real-time PCR primers.
(DOCX)

Table S2 NFATc1 exon-specific PCR primers.
(DOCX)
