# Supplementary figures and images for: Physiological Notch Signaling Maintains Bone Homeostasis via RBPjk and Hey Upstream of NFATc1
Source: PLoS Genet. 2012 Mar 22;8(3):e1002577. doi: 10.1371/journal.pgen.1002577 (PMC3310726; doi:10.1371/journal.pgen.1002577)

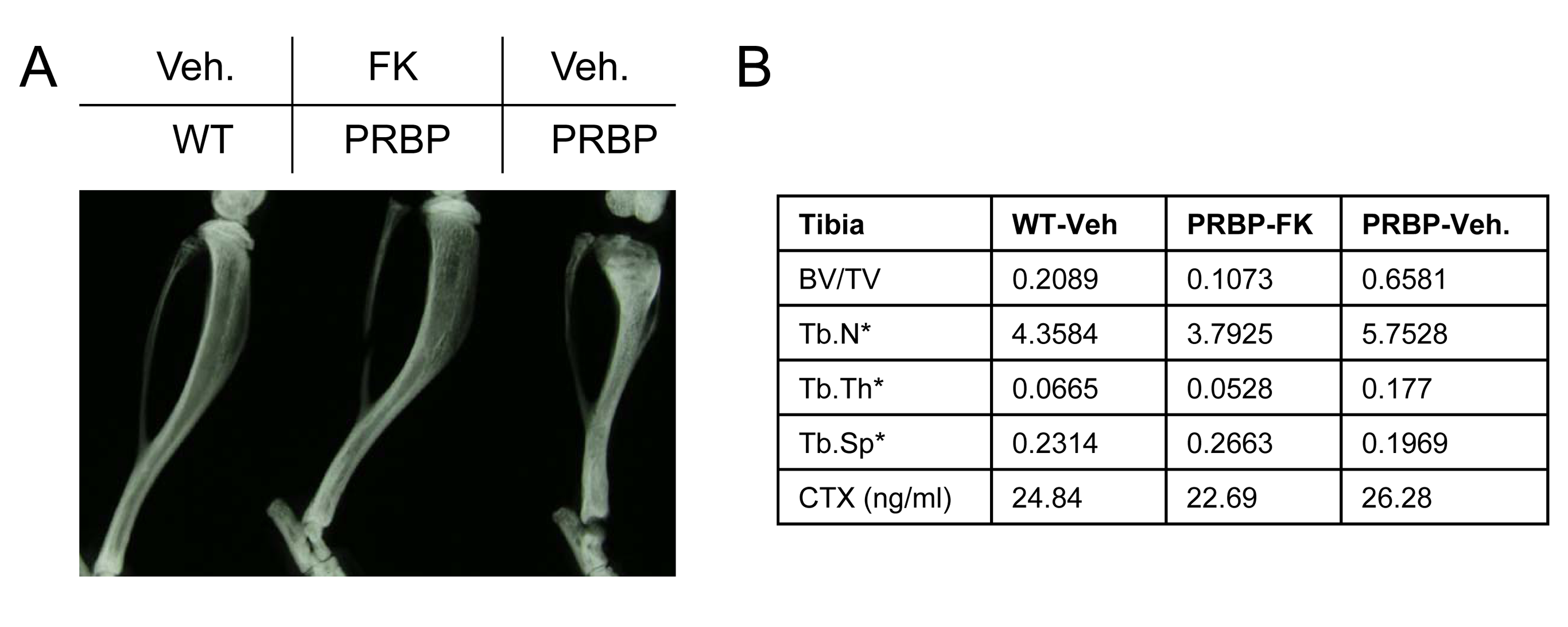

Supplement: Figure S1 — Correction of the high-bone-mass phenotype in the tibia by FK506. (A) X-ray radiographs of the tibia. Note a notable correction of the shape of the tibia. (B) Data from μCT analyses and serum CTX assays of animals in (A). (TIF) [file pgen.1002577.s001.tif]

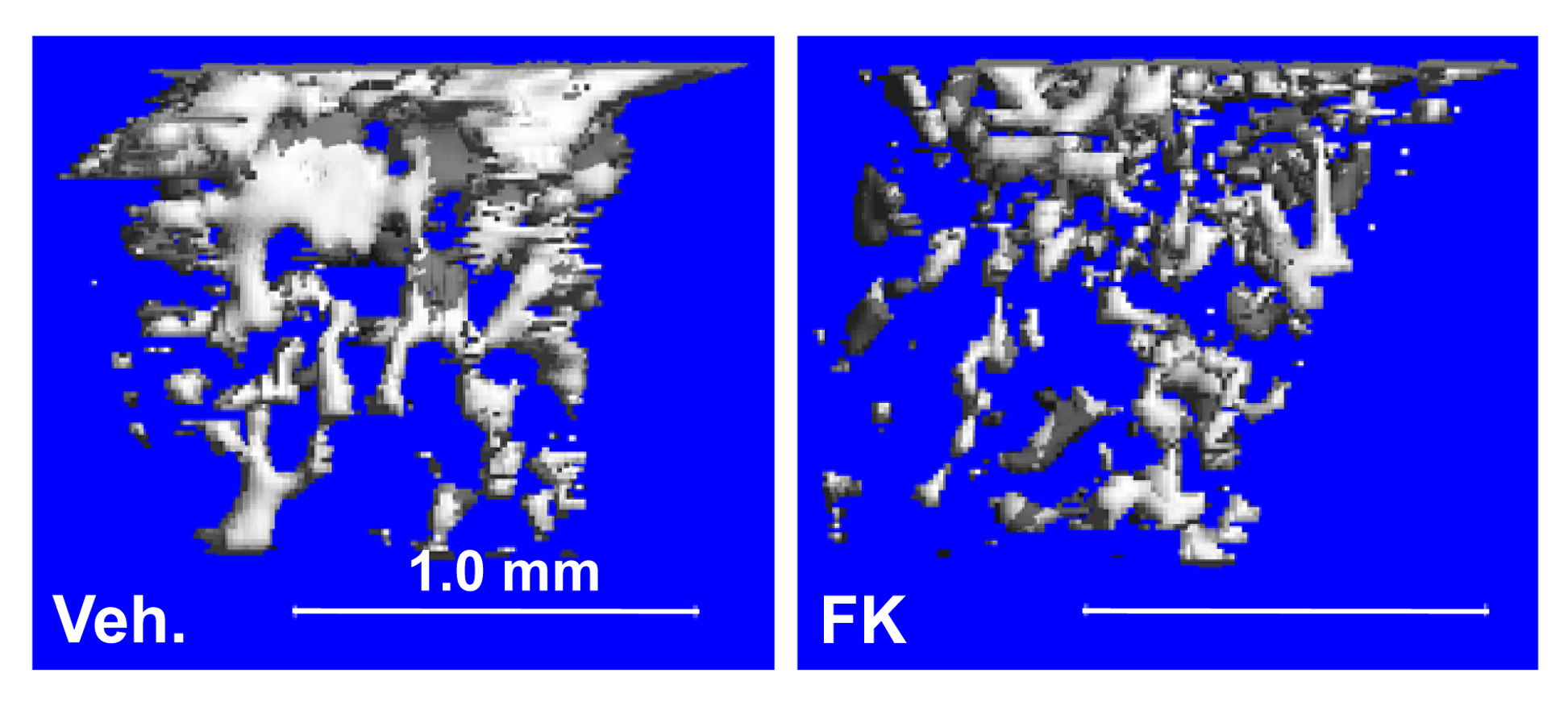

Supplement: Figure S2 — Effect of FK506 on trabecular bone mass of wild type mice. Shown are μCT 3-D reconstruction images of the metaphyseal trabecular region of the tibia. (TIF) [file pgen.1002577.s002.tif]

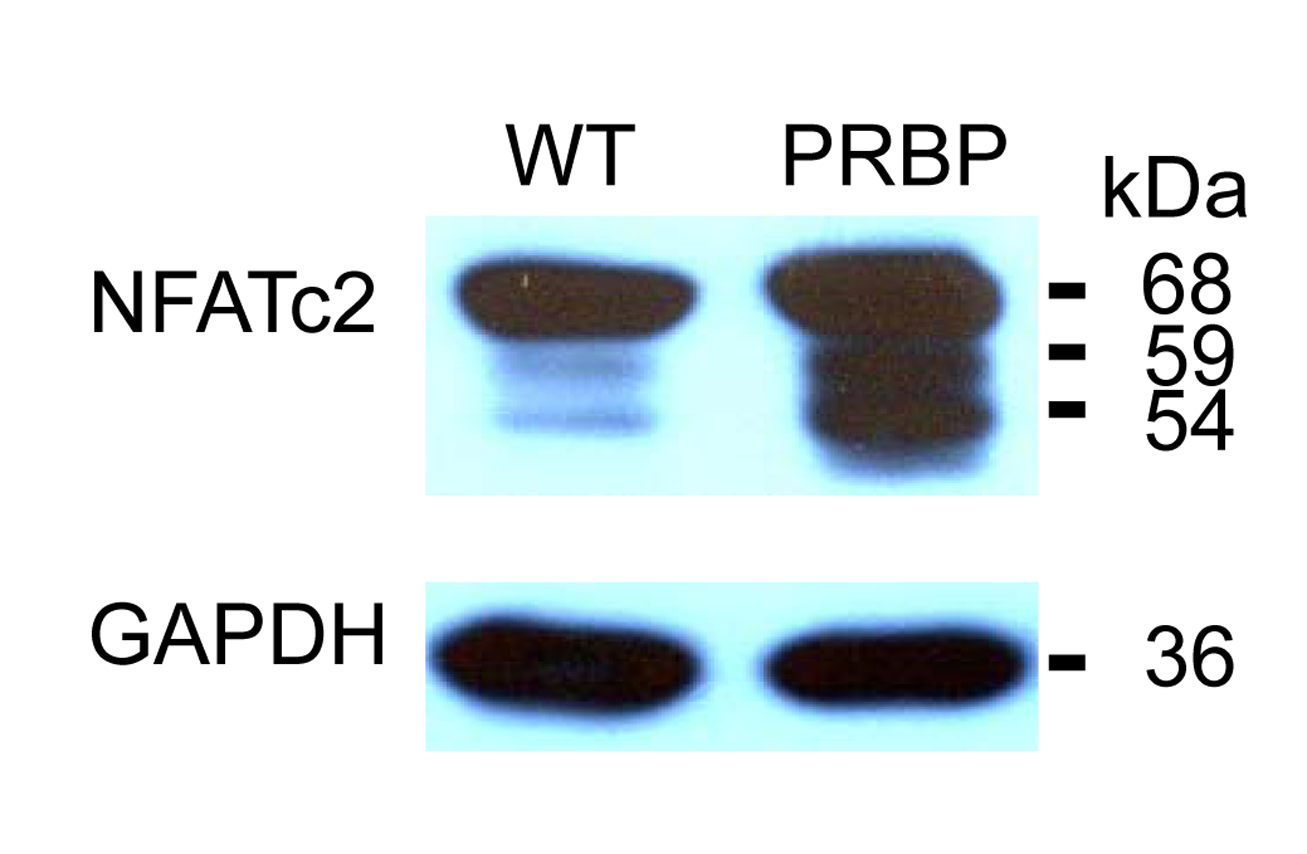

Supplement: Figure S3 — Western analyses of NFATc2 in protein extracts from tibiae and femora of 8-week-old PRBP versus wild-type littermates. (TIF) [file pgen.1002577.s003.tif]

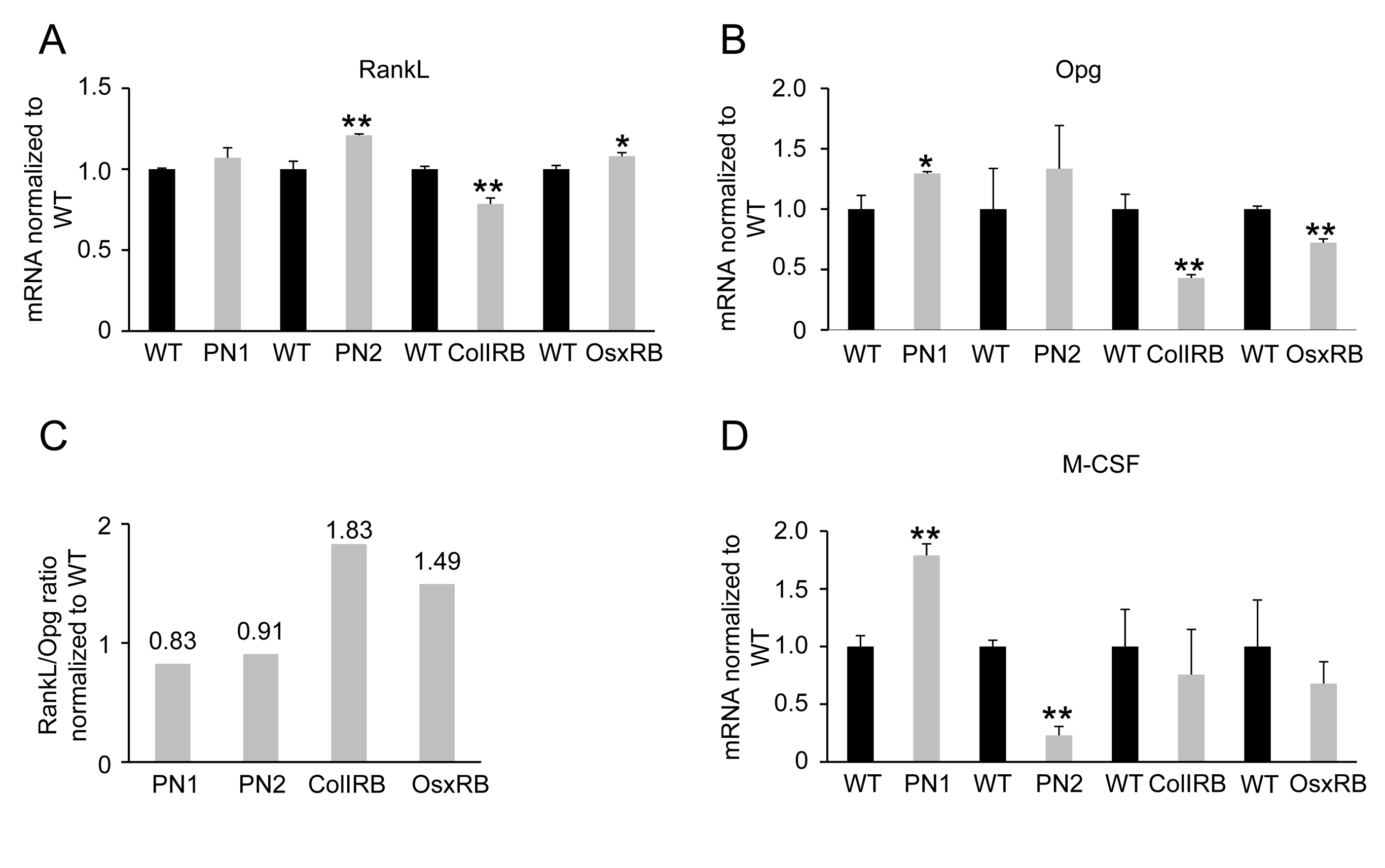

Supplement: Figure S4 — Real-time PCR of osteoclastogenic factors in bone RNA from indicated mouse strains. Values are normalized to wild type levels (designated 1). Bar graphs show mean ± s. d., *p<0.05, **p<0.01, n = 3. (TIF) [file pgen.1002577.s004.tif]
