# Supplementary material for: Physiological Notch Signaling Maintains Bone Homeostasis via RBPjk and Hey Upstream of NFATc1
Source: PLoS Genet. 2012 Mar 22;8(3):e1002577. doi: 10.1371/journal.pgen.1002577 (PMC3310726; doi:10.1371/journal.pgen.1002577)
Supplement: Table S1 — Real-time PCR primers. (DOCX) [file pgen.1002577.s005.docx]

Table S1. Real-time PCR primers

| Gene | Strand | Sequence (5’ to 3’) | Size (bp) |
| --- | --- | --- | --- |
| Hey1 | forward | CACTGCAGGAGGGAAAGGTTAT | 64 |
|  | reverse | CCCCAAACTCCGATAGTCCAT |  |
| HeyL | forward | GAAGCGCAGAGGGATCATAGA | 74 |
|  | reverse | CCAATCGTCGCAATTCAGAA |  |
| Rankl | forward | CTGGGCCAAGATCTCTAACATGA | 118 |
|  | reverse | GGTACGCTTCCCGATGTTTC |  |
| Opg | forward | CCGAGGACCACAATGAACAAGT | 75 |
|  | reverse | CTGGGTTGTCCATTCAATGATG |  |
| M-CSF | forward | CGCTGCCCTTCTTCGACAT | 86 |
|  | reverse | TCTGACACCTCCTTGGCAATACT |  |
| NFATc1 | forward | GAGACAGACATCGGGAGGAAGA | 61 |
|  | reverse | GTGGGATGTGAACTCGGAAGA |  |
| GAPDH | forward | GCACAGTCAAGGCCGAGAAT | 150 |
|  | reverse | GCCTTCTCCATGGTGGTGAA |  |
