# Supplementary material for: Physiological Notch Signaling Maintains Bone Homeostasis via RBPjk and Hey Upstream of NFATc1
Source: PLoS Genet. 2012 Mar 22;8(3):e1002577. doi: 10.1371/journal.pgen.1002577 (PMC3310726; doi:10.1371/journal.pgen.1002577)
Supplement: Table S2 — NFATc1 exon-specific PCR primers. (DOCX) [file pgen.1002577.s006.docx]

Table S2. NFATc1 RT-PCR primers

| Exons | Strand | Sequence (5’ to 3’) | Size (bp) |
| --- | --- | --- | --- |
| 1/3 | forward | ACCAGCTTTCCAGTCCCTTCCAAG | 462 |
|  | reverse | TGCAGGGTTGCTGTAGACGGTG |  |
| 2/3 | forward | GGAGTTCGACTTCGATTTCCTC | 406 |
|  | reverse | TGCAGGGTTGCTGTAGACGGTG |  |
| 8/9b | forward | CTGGGAGATGGAAGCAAAGAC | 347 |
|  | reverse | GCGACTTGGTCTTGTGAATAGGG |  |
| 8/11 | forward | CTGGGAGATGGAAGCAAAGAC | 596 or 885 |
|  | reverse | GTGCTGGAGAGGTCGTTACG |  |
